# Prolonged or serious conflicts at work and incident dementia: a 23-year follow-up of the Copenhagen City Heart Study

**DOI:** 10.1007/s00420-018-1365-9

**Published:** 2018-10-28

**Authors:** Kazi Ishtiak-Ahmed, Åse Marie Hansen, Erik Lykke Mortensen, Anne Helene Garde, Ane Nørgaard, Finn Gyntelberg, Naja Hulvej Rod, Sabrina Islamoska, Rikke Lund, Thien Kieu Thi Phung, Eva Prescott, Gunhild Waldemar, Kirsten Nabe-Nielsen

**Affiliations:** 1 Department of Public Health, University of Copenhagen, Øster Farimagsgade 5, 1014 Copenhagen, Denmark; 2 The National Research Centre for the Working Environment, Lersø Parkallé 105, 2100 Copenhagen, Denmark; 3 Center for Healthy Aging, University of Copenhagen, Blegdamsvej 3B, 2200 Copenhagen, Denmark; 4 Department of Cardiology, Bispebjerg University Hospital, Bispebjerg Bakke 23, 2400 Copenhagen, Denmark; 5 Department of Neurology, Danish Dementia Research Centre, Rigshospitalet, University of Copenhagen Section 6911, Blegdamsvej 9, 2100 Copenhagen, Denmark

**Keywords:** Social relations, Negative aspects of social relations, Psychosocial work factors, Alzheimer’s, Midlife risk factors, Cohort study

## Abstract

**Purpose:**

Only a few studies have investigated the impact of negative aspects of social relations on cognitive function, and they have shown mixed results. Conflicts at work are part of the negative aspects of social relations, but the impact of experiencing conflicts at work has not yet been investigated as a risk factor for dementia. Therefore, we investigated whether experiencing prolonged or serious conflicts with a supervisor or colleagues at work was associated with incident dementia in old age.

**Methods:**

We analyzed data of 6,436 men and women from the third survey of the Copenhagen City Heart Study. At baseline in 1991–1994, the participants reported whether they had ever had a prolonged or serious conflict at work. The participants were followed until 2014. We used Poisson regression to estimate incidence rate ratios (IRR) and their 95% confidence intervals (CI).

**Results:**

After adjusting for potential confounders, the IRR for dementia was 1.53 (95% CI 0.77–3.03) among participants who had reported having prolonged or serious conflicts both with a supervisor and colleagues compared with participants who had never had such conflicts. In separate analyses stratified by sex, the IRRs were 2.14 (95% Cl 0.97–4.71) for men and 0.98 (95% Cl 0.29–3.32) for women.

**Conclusions:**

Our findings did not support an overall association between experiencing prolonged or serious conflicts at work and incident dementia. However, because of the large differences in the point estimates for men and women, future research could aim at investigating potential sex differences regarding the association between conflicts at work and dementia.

## Introduction

A growing body of research has shown a positive effect of social relations in private life on cognitive health (Kuiper et al. 2016), while lack of social relations (low social participation, less frequent social contact and loneliness) has been associated with a higher risk of dementia (Kuiper et al. 2015). Social relations may be a source of support, but may also be a source of relational strain including conflicts and excessive demands (Due et al. 1999). A few studies have investigated the effect of negative aspects of social relations, i.e., conflicts, social strain or negative social interactions causing worries, problems, and stress on cognitive function (Hughes et al. 2008; Liao et al. 2014; Seeman et al. 2001, 2011; Xu et al. 2016). These studies reported mixed results and focused primarily on older populations. None of these studies used clinically diagnosed dementia as outcome. In a study on emotional effects of daily stressors, interpersonal conflicts were identified as the most important stressors, and these conflicts had the strongest adverse effect on a person’s mood (Bolger et al. 1989). Likewise, conflicts at work have also been considered to be severe stressors (Frone 2000; Spector and Bruk-Lee 2008). Thus, experiencing prolonged or serious conflicts at work could be considered as severe stressors.

A potential harmful effect of experiencing prolonged or serious conflicts at work on the development of cognitive impairment or dementia might be due to activation of the physiological stress response (Sherman et al. 2016). Stress increases the release of cortisol, which is known to cause hippocampal atrophy and production/deposition of β-amyloid peptide (Aβ) in the brain (Dong and Csernansky 2009). Deposition of Aβ in the brain is a pathological hallmark of Alzheimer’s disease, which is the most common type of dementia (Dong and Csernansky 2009). A prospective study showed a link between reporting stress in midlife and brain atrophy and white matter lesions in old age (Johansson et al. 2012), which have been suggested to be a mechanism linking midlife psychological stress and risk of dementia in old age (Johansson et al. 2010). Conflicts usually seem to have a temporary effect on health, but if conflicts are poorly managed, the effect could be long term (Carsten et al. 2004). Long-term conflicts are known to be a source of chronic stress (Cohen 2004). Chronic stress is often linked to cardiovascular risk factors and cardiovascular disease (CVD) (Steptoe and Kivimaki 2012). Midlife cardiovascular risk factors, such as blood pressure and cholesterol, have shown to be associated with risk of dementia in later life (Gottesman et al. 2017; Kivipelto et al. 2001). Moreover, being a severe social stressor, experiencing prolonged or serious conflicts may negatively influence individuals’ health behaviors (Cohen 2004; Umberson et al. 2010), which may eventually influence cognitive health. Furthermore, Alzheimer’s disease, the major cause of dementia, has a long pre-clinical phase that can last from a few years to a decade. This means that the underlying brain pathology occurs many years before the disease manifests itself, perhaps already in midlife (40–65 years) (Livingston et al. 2017). Taken altogether, it seems plausible that experiencing prolonged or serious conflicts in midlife may be associated with the risk of dementia later in life.

Experiencing conflicts at work is common. According to a national report in Denmark, one out of five people at the Danish workplaces experiences conflicts at work (The National Research Center for the Working Environment 2016). Interpersonal conflicts at work are associated with poor self-reported general health (De Raeve et al. 2009), poor sleep quality (Fortunato and Harsh 2006), psychiatric morbidity (Romanov et al. 1996), depression (Hagerty and Williams 1999; Meier et al. 2014), and work disability (Appelberg et al. 1996). Despite the high prevalence of conflicts at work (Malgorzata et al. 2009; The National Research Center for the Working Environment 2016) and despite the fact that it is considered a severe social stressor associated with mental disorders, the effect of conflicts at work on the risk of dementia has not yet been investigated. In the current study, we aimed to investigate whether experiencing prolonged or serious conflicts with a supervisor or colleagues at work was associated with incident dementia in old age.

## Methods

### Study population

The study population was drawn from the third survey of the Copenhagen City Heart Study (CCHS) (Aguib and Al Suwaidi 2015). In the first survey of CCHS in 1975, a sample of 19,698 men and women (aged 20 + years) were randomly selected from 90,000 inhabitants of a defined area of central Copenhagen. In the second survey in 1981–1983, the original sample was supplemented with 500 participants. In the third survey in 1991–1994, 3000 participants were added to the previous sample. The third survey formed the baseline in the current study. Out of the 16,563 invited, 10,135 persons agreed to participate. Participants with missing information about conflicts at work (*n* = 1061) were excluded. The excluded participants were older (mean age 70.5 years) than the included participants (mean age 61.2 years), and the majority of them were on an old-age pension.

**Fig. 1:**
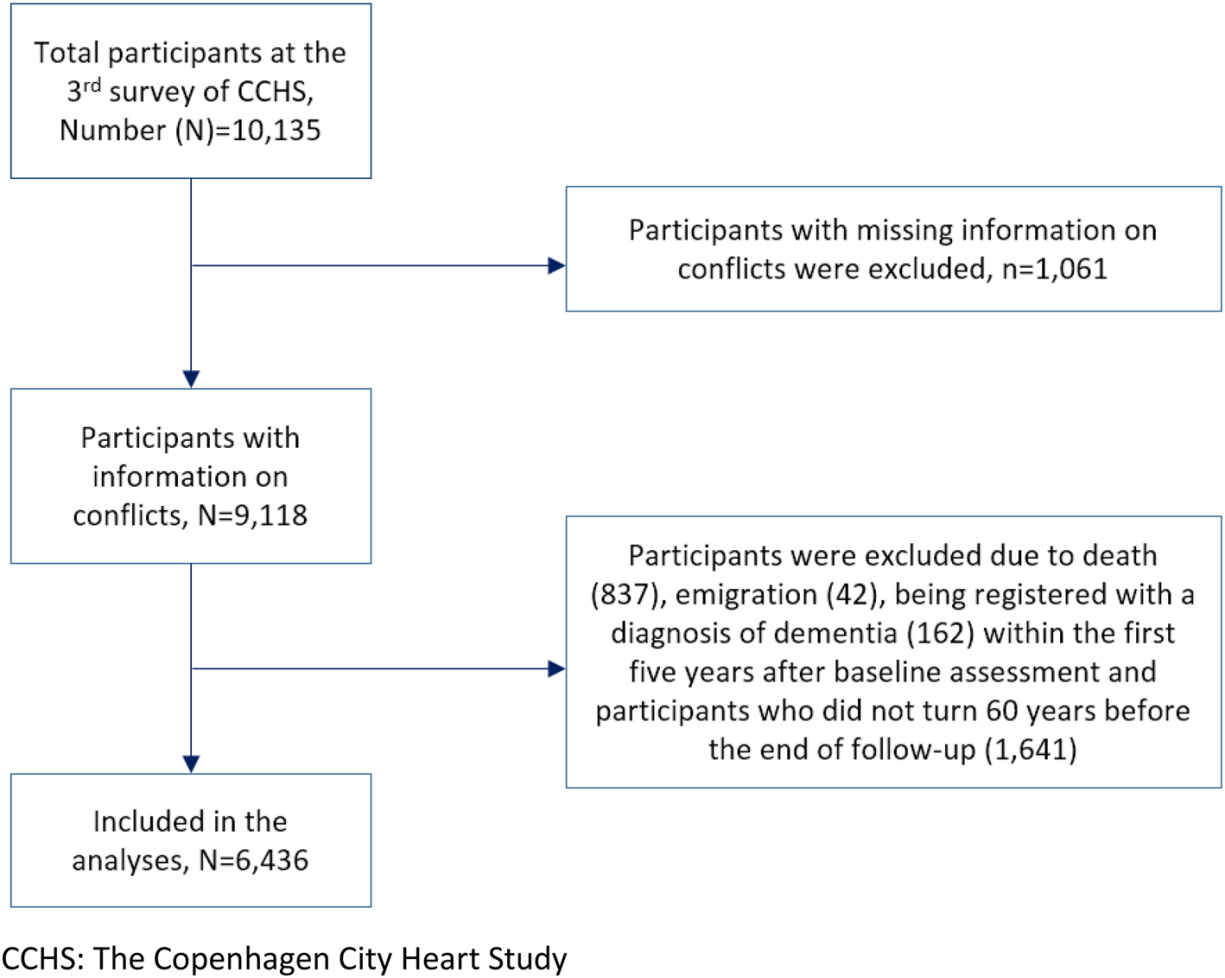
Selection of the study participants for analyses

We started follow-up time when the participants turned 60 years. However, to reduce reverse causality, follow-up time did not start within the 5 years after the baseline assessment in 1991–1994. Applying these two criteria, we were left with 6,436 participants (3666 women) for the analyses, and their mean age was 61.2 years (standard deviation, SD = 11) at baseline. The included participants were followed up until the date of a dementia diagnosis, emigration, death, or to the end of 2014, whichever came first. The flowchart of the selection of the study population is presented in Fig. 1.

### Prolonged or serious conflicts at work

Prolonged or serious conflicts at work were assessed by two questions: (i) “Have you ever had a prolonged or serious conflict with your supervisor?”, and (ii) “Have you ever had a prolonged or serious conflict with your colleagues?” Response options for both questions were “yes/no”.

We analyzed these questions by categorizing the participants into four response groups: participants who (a) never had a prolonged or serious conflict neither with a colleague nor with a supervisor, (b) had a prolonged or serious conflict only with a supervisor, (c) had a prolonged or serious conflict only with a colleague, and (d) had prolonged or serious conflicts with both a supervisor and a colleague.

To experience conflicts with a supervisor and colleagues, participants must have had a job at some point before the baseline. This was verified by linkage to a Danish register with information about employment status (The Employment Classification Module). All of the included participants had been employed at some point between 1976 (start of the register) and the year of the baseline survey.

### Dementia diagnoses

We extracted information on dementia diagnoses from the Danish national registers. List of sources of registers, applied diagnostic codes and the numbers of dementia cases identified are presented in Table 1. Because of the low validity of the dementia subtypes in the registers, we did not differentiate between dementia subtypes in our data analyses (Phung et al. 2007).

**Table 1:** Number of dementia cases identified in the Danish national registers

Dementia sub-types	ICD codes	Number of cases	List of source registers
Alzheimer’s disease	ICD-8: 290.09; ICD-10: F00.0-9, G30.0-9	214	The Danish Psychiatric Central Research Register (Munk-Jorgensen and Mortensen 1997) The Danish National Patient Register (Andersen et al. 1999) and The Danish Register of Causes of Death (Helweg-Larsen 2011)
Vascular dementia	ICD-8:293.09-19; ICD-10:F01.0-9	68	
Frontotemporal dementia/dementia with Lewy bodies	ICD-8: 290.11; ICD-10: F02.0/ ICD-10: G31.8	23^b^	
Dementia without specification	ICD-8: 290.09; ICD-10:F03.9, G31.9	440	
Total number of dementia cases		745^a^	

*ICD* International Classification of Diseases

^a^There were nine cases that had double sub-types

^b^The vast majority were dementia with Lewy bodies (Statistics Denmark does not provide data with a frequency of ≤ 3, which is the case of frontotemporal dementia; therefore, we obtained a combined figure for both subtypes)

### Covariates

From the national registers in Denmark, we obtained information about the participants’ age, sex, educational attainment (0–9 years/11–12 years/>12 years of formal schooling), co-morbidities before baseline including register-based diagnoses of diabetes and CVD (i.e., any ischemic heart disease and cerebrovascular disease, yes/no), and psychiatric disorders (record of hospitalization in a psychiatric department/hospital before baseline).

In the questionnaire, the participants were asked about the frequency of contact with parents, children, other family members, meeting colleagues outside of working hours, neighbors, childhood and youth friends, other friends, acquaintances, and house assistants. They were asked about their satisfaction with these contacts (response options: very much/more or less, or not at all) and whether they could talk about something personal with any of these contacts (i.e., whether they had a confidant). We used the latter two questions as two separate variables in our analyses as a measure of social relations in private life.

Information about lifestyle/CVD-risk factors was obtained from questionnaires: (i) smoking (current smoker/past smoker/never smoker), (ii) physical activity in leisure time (almost sedentary life/light physical activity/moderate physical activity or vigorous physical activity), (iii) the average unit of alcohol consumption per week (beer, glass of wine, spirits), and (iv) use of sleeping pills.

From the clinical examination, we obtained information on the participants’ body mass index (BMI) = (weight in kg)/(height in meter)^2^, and systolic blood pressure (mmHg). Age, average alcohol consumption per week, BMI, and systolic blood pressure were analyzed as continuous variables.

### Statistical analyses

To examine the association between prolonged or serious conflicts at work and incident dementia in old age, we used Poisson regression analyses and estimated incidence rate ratios (IRR) and their 95% confidence intervals (95% CI).

In the multivariable analyses, we adjusted for covariates that are likely to be potential confounders of the association between conflicts at work and dementia. In model 1, we adjusted for current age, sex, time since baseline assessment, and calendar year. In model 2, we further included participants’ educational attainment, psychiatric disorders, and private life social relations (Livingston et al. 2017; Stein et al. 2008). In model 3, we also included lifestyle/CVD-risk factors (smoking, physical activity in leisure time, alcohol consumption, BMI, systolic blood pressure and use of sleeping pills), and comorbidities (diabetes, CVD).

The group of covariates included in model 3 could be confounders as well and mediators of the investigated relationship between conflicts at work and dementia (Rod et al. 2009; Stein et al. 2008; Umberson et al. 2010). From the available information in our data, we are not certain about the temporal relationship between experiencing prolonged or serious conflicts at work and any changes in lifestyle/CVD-risk factors. Therefore, we considered model 2 as our main model.

A total of 204 (3.2%) participants had missing information on at least one variable (satisfaction with private life social relations, *n* = 70; smoking, *n* = 1; physical activity during leisure time, *n* = 18; alcohol, *n* = 45; BMI, *n* = 54; blood pressure, *n* = 43, and use of sleeping pills, *n* = 26). Participants with missing information were excluded analysis-by-analysis. A loss to follow-up due to emigration was minimal (0.6%).

### Supplementary analyses

In a sensitivity analysis, we used a dichotomized explanatory variable (prolonged or serious conflicts at work with a supervisor and/or colleagues: yes/no) instead of four categories. To explore the differences between men and women, we stratified our analyses by sex in another sensitivity analysis. Both sensitivity analyses were carried out by rerunning the main model (model 2).

We performed all our statistical analyses in SAS version 9.2.

## Results

A total of 736 (11.4%) participants were diagnosed with dementia during an average of 17.2 years of follow-up. The mean age at the time of dementia diagnosis was 82.4 (SD 7.1) years.

In Table 2, baseline characteristics of the study population are presented. In general, the four response groups did not differ substantially in terms of their baseline characteristics. However, participants who reported conflicts were considerably younger, had lower systolic blood pressure, were less satisfied with their private life social relations, and were better educated compared with those reporting no conflicts.

**Table 2:** Baseline characteristics of the study population (*N* = 6436)

Characteristics	Prolonged or serious conflicts at work			
	Never *N* = 5883	Only with supervisor *N* = 279	Only with colleague, *N* = 157	With supervisor and colleague, *N* = 117
Outcome				
Dementia cases during follow-up, *N* (%)	696 (11.8)	18 (6.5)	13 (8.3)	9 (7.7)
*Baseline characteristics*				
Socio-demographic factors				
Age (years), mean (SD)	61.8 (10.9)	55.1 (9.4)	53.9 (10.2)	53.2 (9.7)
Women, *N* (%)	3395 (57.7)	120 (43.0)	92 (58.6)	59 (50.4)
Follow-up (years), mean (SD)	17.1 (5.4)	19.1 (4.6)	19.7 (3.7)	19.0 (4.9)
Potential confounders				
Educational attainment: ≤ 9 years, *N* (%)	2867 (48.7)	74 (26.5)	38 (24.2)	29 (24.8)
Satisfied with private life social relations: more or less/not at all, *N* (%)	1729 (29.7)	113 (41.1)	65 (41.7)	57 (48.7)
Have a confidant: no, *N* (%)	416 (7.1)	19 (6.8)	11 (7.0)	10 (8.6)
Psychiatric disorders: yes, *N* (%)	361 (6.1)	26 (9.3)	19 (12.1)	8 (6.8)
Potential mediators				
Diabetes/CVD: yes, *N* (%)	391 (6.7)	15 (5.4)	7 (4.5)	Few obs.^a^
Smoking: current smoker, *N* (%)	2857 (48.6)	137 (49.1)	63 (40.1)	63 (53.9)
Physical activity during leisure time: almost sedentary life, *N* (%)	658 (11.2)	29 (10.4)	15 (9.6)	15 (12.9)
Alcohol (average unit per week), mean (SD)	9.2 (12.0)	13.0 (14.2)	9.9 (11.3)	11.2 (11.3)
BMI (kg/m^2^), mean (SD)	26.0 (4.3)	25.7 (4.3)	25.5 (3.9)	25.5 (4.7)
Systolic blood pressure, mean (SD)	142 (21)	135 (20)	129 (19)	130 (20)
Takes sleeping pills: yes, *N* (%)	478 (8.2)	15 (5.4)	9 (5.7)	6 (5.1)

^a^Few obs.—due to a restriction from the Statistics Denmark, we were not allowed to present any information which contains a frequency ≤ 3

Overall, we did not find significant associations between conflicts at work and dementia in any of the models (Table 3). In model 2, the IRR for participants reporting prolonged or serious conflicts with a supervisor and colleagues was 1.53 (95% Cl 0.77–3.03) compared with participants reporting no such conflicts. In model 3, the estimate remained essentially the same when adjusting for potential mediators. The IRR for participants who had prolonged or serious conflicts either with a supervisor or with colleagues did not differ from unity, and the IRRs were 0.90 (95% Cl 0.56–1.46) and 1.06 (95% Cl 0.58–1.94), respectively, in model 2.

**Table 3:** Incidence rate ratios (IRR) with 95% confidence interval (CI) for incident dementia in participants according to experiencing prolonged or serious conflicts at work

	No. of dementia cases	Univariate analysis	Multivariable analyses		
	*N* = 736/6,436 (11.4%)	IRR (95% Cl)	Model 1 IRR (95% Cl)	Model 2 IRR (95% Cl)	Model 3 IRR (95% Cl)
Never had conflict	696/5883 (11.8%)	1	1	1	1
Only with supervisor	18/279 (6.5%)	0.53 (0.33–0.84)	0.87 (0.54–1.41)	0.90 (0.56–1.46)	0.84 (0.51–1.38)
Only with colleague	13/157 (8.3%)	0.71 (0.42–1.21)	0.98 (0.54–1.78)	1.06 (0.58–1.94)	1.12 (0.61–2.06)
Conflicts with supervisor and colleague	9/117 (7.7%)	0.74 (0.39–1.41)	1.45 (0.73–2.88)	1.53 (0.77–3.03)	1.55 (0.78–3.10)

Model 1: adjusted for current age, sex, time since baseline assessment and calendar year

Model 2: model 1 + educational attainment, private life social relations, psychiatric disorders

Model 3: model 2 + comorbidities (CVD and diabetes), and lifestyle/CVD-risk factors (smoking, physical activity during leisure time, alcohol, BMI, blood pressure, and use of sleeping pills)

When we treated the explanatory variable as dichotomous in a sensitivity analysis, the association between experiencing conflicts at work and incident dementia became negligible with an IRR of 1.05 (95% Cl 0.75–1.48) (not shown in tables).

Results from the second sensitivity analysis showed that the estimated differences (IRRs) were larger in men than in women (Table 4). The adjusted IRR for men who reported having had prolonged or serious conflicts both with a supervisor and colleagues was 2.14 (95% Cl 0.97–4.71), but for women, the IRR was 0.98 (95% Cl 0.29–3.32). Yet, the 95% CIs are broad and overlapping.

**Table 4:** Incidence rate ratios (IRR) with 95% confidence interval (CI) for incident dementia according to sex

Explanatory variable	Men (*N* = 2770), IRR, (95% Cl)	Women (*N* = 3666), IRR, (95% Cl)
Never had conflict	1	1
Only with supervisor	1.06 (0.60–1.90)	0.66 (0.27–1.60)
Only with colleague	1.25 (0.55–2.82)	0.91 (0.37–2.23)
Conflicts with both	2.14 (0.97–4.71)	0.98 (0.29–3.32)

Adjusted for current age, time since baseline assessment, calendar year, educational attainment, private life social relations and psychiatric disorders

## Discussion

### Main findings

In this prospective population-based study of 6,436 Danish middle-aged men and women, we found no overall association between experiencing prolonged or serious conflicts at work and incident dementia in old age. The IRR for the association between conflicts at work and dementia was considerably larger in men than in women. However, these results are based on few cases and with overlapping CI.

### Comparison with previous studies

To the best of our knowledge, no previous studies have investigated the association between conflicts at work and the risk of dementia. In most cases, clinical dementia is preceded by a decline in cognitive function, and although a decline in cognitive function does not always lead to a dementia diagnosis (Richard and Brayne 2014); we also include results from studies of cognitive functioning in the following discussion.

Our findings are in contrast to results from two previous studies that reported an adverse effect of negative aspects of social relations on cognitive function (Liao et al. 2014; Xu et al. 2016). Yet, another study showed no effect (Seeman et al. 2001), and another one reported better cognitive function among participants reporting higher social interactions (Hughes et al. 2008). Similar to our study, some of these past studies estimated negative social relations in midlife (Liao et al. 2014; Seeman et al. 2011). Nevertheless, the contradictions between our findings and findings from past studies suggest a differential role of social relations at work versus social relations in private life on cognitive function. Social relations in private life could be more important to people, and the impact may often be more prolonged, whereas people are likely to leave their job if they experience major conflicts at work.

Our findings raise the question whether there is a sex difference in the association between conflicts at work and dementia. Having conflicts with a supervisor and colleagues appear to be associated with a higher risk of dementia among men, although, none of the estimates were statistically significant and the results were based on few cases. Only a few studies stratified their analyses by sex (Liao et al. 2014; Seeman et al. 2001). However, these studies did not reveal any difference between men and women regarding the association between negative social relations and cognitive function. Because of gender segregation (both horizontal and vertical) at the Danish labor market in the 1980s and 1990s (Bloksgaard 2011), the psychosocial working environment may have differed between male- and female-dominated jobs. Such differences may partly explain the difference between men and women regarding the association between conflicts at work and dementia. However, we cannot support this hypothesis with empirical data, as we do not have information about the participants’ working environment.

We hypothesized that as a severe stressor, conflicts at work might be linked to the risk of dementia in old age through various potential mechanisms. For example, conflicts could cause prolonged stress that leads to brain atrophy and Aβ deposition in the brain, which are considered hallmarks of Alzheimer’s disease. Prolonged stress also influences cardiovascular health, mental health and health-related behaviors, which could eventually lead to dementia. However, our data did not support our hypothesis. There may be several reasons for the negative results, including the possibility that (1) the follow-up time was too long resulting in a dilution of the effect, (2) we had too few cases, (3) conflicts were only assessed at one point in time, (4) the applied questions assessing conflicts at work were insufficient to assess actual conflicts, or (5) that our hypothesis was not valid.

### Strengths and limitations of the study

The main strength of our study is the long-term follow-up and the exclusion of the first 5 years of follow-up after the baseline assessment, which was done to reduce the risk of reverse causation, although reverse causation might still be a concern (Sperling et al. 2011). Nevertheless, with the long follow-up time, the association is likely to be diluted. This might be difficult to avoid when investigating long-term effects of midlife exposures on an outcome occurring primarily in old age, such as dementia. An additional strength of the current study is the adjustment for a wide range of covariates including various indicators of private life social relations, e.g., satisfaction with private life social relations and having a confidant or not. Furthermore, there was a negligible loss to follow-up due to emigration.

One of the main limitations of the present study is the low prevalence of participants who reported prolonged or serious conflicts at work (8.5%). The prevalence was somewhat lower than observed in a recent survey in Denmark, in which conflicts at work were operationalized as “experiencing any quarrels or conflicts at work within the past 12 months” (The National Research Center for the Working Environment 2016). Conflicts at work have been investigated within the past decades, within the areas of organizational management and occupational medicine. Some scales for assessing conflicts at work were developed in the 1980s and 1990s, including the Interpersonal Conflict at Work Scale, the Organizational Constraints Scale, and the Quantitative Workload Inventory (Spector and Jex 1998). In our data, conflicts were assessed by two questions, and our measures might not include all the variations that the more comprehensive scales capture. Future studies should investigate the association between conflicts at work and dementia using a validated scale. As our measures of conflicts at work did not address the number, duration, severity, and timing of conflicts at work, we are likely to miss information that is important for the hypothesized association between these conflicts and dementia risk. Also, our measures cannot differentiate between the duration and severity of conflicts, as it combines “prolonged” and “serious” conflicts as one entity although the two could have different effects. Another limitation related to our measures of conflicts is that they were assessed at only one point in time. This could have led to exposure misclassification, which may have diluted the observed association between conflicts and dementia.

Furthermore, self-reported information on conflicts at work and private life social relations can be influenced by individual characteristics. For instance, people who score high on the personality trait neuroticism may report more conflicts and negative social relations. According to a recent review, neuroticism has been shown to increase the risk of dementia, while conscientiousness protects against dementia (Low et al. 2013). Moreover, other studies have suggested that neuroticism increases the risk of interpersonal conflicts at work (Appelberg et al. 1991) and decreases the number of confidants (Kendler et al. 2003). Therefore, our results could be confounded by unmeasured personality factors. Lifestyle-related factors, e.g., smoking, physical activity during leisure time, and alcohol consumption, can reflect people’s behavior and thereby also certain personality traits. However, we cannot confirm this hypothesis with our data. Adjustment for lifestyle-related factors in our analysis, however, did not change the results substantially.

Among other unmeasured confounders, work-related factors (job control and job complexity) and cognitive ability at baseline may have influenced our results. Low job control and low job complexity in midlife are associated with lower cognitive function and a higher risk of dementia in old age (Then et al. 2014). We adjusted for educational attainment in our analyses, as educational attainment influences what type of occupation a person will have. Furthermore, a recent study suggested occupational complexity strongly mediates the cognitive gain associated with higher levels of education (Fujishiro et al. 2017). Also, educational attainment has been shown to be strongly correlated with cognitive ability in both young adulthood and later life (Mortensen et al. 2014). Yet, adjustment for education did not considerably change the results in the present study. Therefore, it is unlikely that lack of adjustment for job complexity and baseline cognitive ability has substantially influenced our results. Furthermore, we did not have full information on the number of years of employment before baseline, which could be a confounder and may have affected our results.

We adjusted for aspects of social relations in private life in our analyses, but not for other aspects of social relations at work than prolonged or serious conflicts. Both low social support at work (Andel et al. 2012) and a lack of contact with colleagues during working hours (Ishtiak-Ahmed et al. 2017) have been shown to be associated with higher risk of dementia. The buffering effect of social support on stressful life events in relation to health outcomes is well documented in the literature (Umberson et al. 2010). Also, individuals with social support at work may react differently to prolonged or serious conflicts at work compared with individuals without social support.

A validation study reported that the positive predictive value of the registered dementia diagnoses in the Danish national hospital registers was 86% (Phung et al. 2007). However, the hospital registers only capture about two-thirds of all cases in Denmark (Phung et al. 2010). This underreporting could have affected our results by diluting the association between conflicts and dementia in the present study. It is unlikely that missing information has influenced our results as only 1.1% had at least one variable with missing information among the covariates included for adjustments in the main analyses.

Another concern is that 10% of the 10,135 participants in the third survey of CCHS did not respond to the questions on conflicts at work. Non-responders have increased mortality and morbidity in most epidemiological studies (Rothman et al. 2008). If they had had more conflicts at work compared to responders, it can blunt our results. The percentages of dementia among the excluded and included participants were 16% and 11% respectively, and the incidence rates were 156 and 119 per 10,000 person-years, respectively. Furthermore, among the 837 participants (mean age: 70 years) who were excluded due to death within the first 5 years since baseline assessment, 4.2% of them reported experiencing conflicts at work compared to 8.5% of the included participants, indicating that the participants’ status of reporting conflicts may not have influenced these deaths. Thus, the exclusion based on mortality within five years from baseline are unlikely to affect our results.

## Conclusions

This is the first prospective study investigating the impact of conflicts at work on the risk of dementia in old age. Our data did not support an association between experiencing prolonged or serious conflicts at work and the risk of dementia. However, our results were based on a few cases. Because of the large difference between the point estimates for men and women in our sensitivity analyses, we cannot draw firm conclusions on sex difference, but it may be relevant for future studies to explore sex differences in the association between conflicts at work and incident dementia.

## Abbreviations

IRR: Incidence rate ratios
CI: Confidence interval
Aβ: β-Amyloid peptide
CCHS: The Copenhagen City Heart Study
PPV: Positive predictive value
SD: Standard deviation
ICD: International classification of diseases
CVD: Cardiovascular disease
BMI: Body mass index
